# A Six-Year Longitudinal Study of Psychological Distress, Depression, Anxiety, and Internet Addiction Among Students at One Medical Faculty

**DOI:** 10.3390/healthcare13141750

**Published:** 2025-07-19

**Authors:** Meltem Akdemir, Yonca Sonmez, Yesim Yigiter Şenol, Erol Gurpinar, Mehmet Rifki Aktekin

**Affiliations:** 1 Department of Public Health, Akdeniz University Faculty of Medicine, Antalya 07058, Turkey; yoncasonmez@akdeniz.edu.tr (Y.S.); maktekin@akdeniz.edu.tr (M.R.A.); 2 Department of Medical Education, Akdeniz University Faculty of Medicine, Antalya 07058, Turkey; yigiter@akdeniz.edu.tr (Y.Y.Ş.); erolgurpinar@akdeniz.edu.tr (E.G.)

**Keywords:** longitudinal study, medical students, depression, anxiety, psychological distress

## Abstract

**Background**: Medical education is considered one of the most academically and emotionally demanding training programs. Throughout their education, medical students are exposed to various factors that can lead to psychological distress, depression, and anxiety. The aim of this longitudinal study was to examine the changes in psychological distress, depression, anxiety levels and internet addiction among medical students throughout their six-year education and to identify the contributing factors. **Methods**: The study cohort consisted of 282 students who enrolled in the medical faculty in the 2017–2018 academic year. A questionnaire including sociodemographic characteristics, the General Health Questionnaire-12 (GHQ-12), Beck Depression Inventory (BDI), State–Trait Anxiety Inventory (STAI), and Young Internet Addiction Test (IAT) was administered to the students during the first week of their education. The same questionnaire was readministered at the end of the third and sixth years. Friedman’s variance analysis was used to compare measurement data across the three time points, while Cochran’s Q Test was employed for categorical variables. **Results**: The median scores of the GHQ-12, BDI, S-Anxiety, and IAT significantly increased from the first to the sixth year (*p* < 0.05). The prevalence of depressive symptoms, S-Anxiety, and risky internet use significantly increased from the first to the final year, particularly between the third and sixth years. According to logistic regression analysis based on sixth-year data, students whose fathers were university graduates, who had been diagnosed with COVID-19, and who were dissatisfied with their social lives were found to be at increased risk for psychological distress and depression. Students with high parental expectations were found to be at risk of depression and S-anxiety. Those dissatisfied with their occupational choice were at risk for both psychological distress and S-anxiety. **Conclusions**: It was found that the mental health of medical students deteriorated during their education, especially during the clinical years. Given that these students will be responsible for protecting and improving public health in the future, it is essential to prioritize their own mental well-being. Interventions aimed at preserving the mental health of medical students should be planned.

## 1. Introduction

Mental health problems are highly prevalent worldwide. According to the World Health Organization, while approximately one in eight people globally lived with a mental disorder in 2019, this number increased further in 2020 due to the COVID-19 pandemic. Among mental disorders, depressive and anxiety disorders are the most common [1].

A range of individual, familial, social, and structural factors can either support or undermine mental well-being. Schools and workplaces can represent both opportunities and risks for mental health. Adverse working environments or negative life events are known to increase the risk of developing depression and anxiety disorders. Certain periods in life are more critical than others in this regard [1,2]. University life is one such critical period, encompassing the transition from adolescence to adulthood. During this phase, individuals often strive for independence from their families and experience significant changes in social roles. Instability may be observed in peer and romantic relationships, as well as in academic or career choices [3,4,5]. While navigating this period of self-discovery, students may encounter various challenges within the university setting. These instabilities and difficulties can lead to increased stress, which is a known contributing factor to the development of mental disorders [3]. Studies have shown that mental health problems such as depression and anxiety are common among university students, with 12–50% reported to have one or more mental disorders [3,4]. There is also evidence that medical students are more likely to experience mental health problems than their peers in the general population and other academic fields [6,7,8,9,10]. Recent meta-analyses indicate that the prevalence of depression among medical students is 28.0% (95% CI: 24.2–32.1%) and the prevalence of anxiety is 33.8% (95% CI: 29.2–38.7%), suggesting that approximately one in three medical students suffers from significant psychological distress. Some longitudinal studies have also concluded that medical education adversely affects students’ mental health over time [7,11,12].

Throughout their education, medical students are exposed to various factors that can lead to psychological distress, depression, and anxiety. These factors include the difficulty of medical training, the prolonged duration of education, an intensive curriculum, frequent examinations, limited allowances for absenteeism, sleep deprivation, long working hours, excessive workload, a competitive environment, dissatisfaction with their medical education or career choice, financial difficulties, institutional factors, patient contact, and exposure to patient deaths [6,10,11,12,13,14,15,16].

Mental health problems can negatively impact medical students’ quality of life, social functioning, and academic performance and may even lead to dropping out of medical school [17]. Studies have reported that students experiencing depression and anxiety tend to show lower levels of empathy and are less inclined to provide care for patients with chronic illnesses in the long term [10,11].

The aim of this study is to examine the changes in psychological distress, depression, anxiety levels, and internet addiction among medical students throughout their six-year education and to identify the contributing factors.

## 2. Materials and Methods

### 2.1. Setting and Sample Description

This study is a longitudinal follow-up study. The research cohort consisted of all the students (*n* = 282) who enrolled in the Akdeniz University Faculty of Medicine in the 2017–2018 academic year. The Akdeniz University Faculty of Medicine is located in south of Türkiye. The number of students who were included in the study and agreed to participate was 282 (100.0%) in the first year, 259 (91.8%) in the third year, and 230 (81.6%) in the sixth year.

### 2.2. Data Collection

The research data were collected through a self-administered paper-based questionnaire. The questionnaire prepared by the researchers based on a literature review was administered to the students during the first week of school. The same questionnaire, with some additional questions, was re-administered to the students at the end of the third and sixth years. Ten students dropped out of medical school after one year, five students transferred to different medical schools in the country, and thirty-seven students continued their education at the same school but did not want to answer the questionnaire. The total number of students missing in the study was 52 (18.4%). To minimize follow-up losses, questionnaires were administered to students during their non-exam periods.

Medical education in Türkiye lasts six years. The first three years are known as the preclinical period and are mostly composed of theoretical courses. Starting from the fourth year, students begin hospital clerkships. The sixth year is the internship period and is spent entirely on patient examinations and follow-up, with students taking on more clinical responsibility. Akdeniz University Faculty of Medicine has a program model that is integrated based on organ systems in the first three years (horizontally integrated), where basic and clinical sciences are associated through problem-based learning (vertically integrated), and also frequently includes educational goals for society. In the 4th and 5th years, there is horizontal and vertical integration based on clinical clerkships, where basic sciences are less important and clinical sciences are more important. There are a total of 19 clerkships in the 4th and 5th years. During this period, the program includes patient-side practices/applications and oral exams that students have not encountered before [18].

The questionnaire included questions on students’ sociodemographic characteristics (age, gender, income status, family type, educational level of parents, presence of psychiatric illness in the family, whether they chose to study medicine willingly, etc.); smoking, e-cigarette, and alcohol habits; regular exercise habits; daily sleep duration; and life events that may cause stress. In addition, the General Health Questionnaire-12 (GHQ-12), Beck Depression Inventory (BDI), State–Trait Anxiety Inventory (STAI), and Young Internet Addiction Test (IAT) were used. Due to the global pandemic and the devastating earthquake that occurred in Türkiye in 2023 during the follow-up period, additional questions were included in the sixth-year questionnaire regarding whether the student had been diagnosed with COVID-19, whether a close relative and/or friend had died due to COVID-19 during the pandemic, and whether a close relative and/or friend was present in the earthquake-affected area.

The 12-item GHQ was used because it provides information on general mental health problems. A 0-0-1-1 scoring system was used for each item to calculate the prevalence of psychological distress. With this scoring system, the minimum score is 0 and the maximum is 12. In prevalence calculations, a cut-off score of 2 points, determined in the Turkish validity and reliability study for GHQ-12, was used, and scores of 2 and above were considered indicative of the presence of psychological distress. Additionally, as another frequently used method, the items were scored between zero and three (Likert method), and mean scores were calculated. According to this scoring method, the minimum score was 0 and the maximum was 36 [19,20].

Depressive symptoms were evaluated using the 21-item BDI. Each item was scored between 0 and 3, such that the minimum total score was 0 and the maximum was 63, with higher scores indicating greater levels of depression. In prevalence calculations, a cut-off point of 17, established in the Turkish validity and reliability study, was used [21,22].

Anxiety levels were assessed using the STAI, which consists of two separate 20-item scales. The state anxiety (S-Anxiety) scale determines how the individual feels at a given moment under specific conditions. The trait anxiety (T-Anxiety) scale indicates how the individual generally feels, regardless of circumstances. Each item is scored between 1 and 4. The total score obtained from each scale ranges between 20 and 80, with higher scores indicating higher anxiety levels. In the literature, a cut-off score (39/40) has been defined for S-Anxiety, but not for T-Anxiety. Both anxiety scales have Turkish validity and reliability studies [23,24,25].

To measure internet addiction in this study, the 20-item Internet Addiction Test (IAT) developed by Young was used. Each item is scored between 0 and 5. The total score ranges from 0 to 100. A higher score indicates higher addiction. A total score of 80 and above indicates “internet addiction/severe IA level,” a score between 50 and 79 indicates “risky internet use/moderate IA level,” and a score of 49 or below is classified as an “average internet user” not experiencing problems due to internet use. The scale has a Turkish validity and reliability study [26,27].

Some events that may be considered stress-inducing—such as financial problems, personal or societal concerns about the future, high expectations from/of the family, dissatisfaction with social life, communication problems with the opposite sex, dissatisfaction with occupational choice, perceived academic success concerns, worry about the adequacy of education, exposure to negative attitudes from faculty members, preparation for the medical specialty exam, and compulsory service after graduation—were assessed using 12 items. Each item was scored by students between 0 (no stress at all) and 10 (very high stress) based on the perceived level of stress caused by the item.

### 2.3. Ethical Considerations

This study complies fully with the principles of the Declaration of Helsinki. All students were informed about the research and were included after giving verbal and written informed consent. Administrative permission and approval were obtained from the Clinical Research Ethics Committee of Akdeniz University for the study (280–10 May 2017).

### 2.4. Statistical Analyses

Analysis of the study data was performed using the Statistical Package for the Social Sciences (SPSS) for Windows (IBM Corp., Armonk, NY, USA) version 23.0. Descriptive statistics of the variables were presented as numbers, frequencies, means, medians, and standard deviations. The normality of data distribution was evaluated using the Kolmogorov–Smirnov test, and the data were found not to be normally distributed. To compare the measurement data obtained at three different time points during follow-up, Friedman’s variance analysis was used, and to determine the source of differences, the Wilcoxon signed ranks test with Bonferroni correction was applied. Cochran’s Q test and McNemar’s test were used to determine time-dependent differences in categorical variables. Additionally, using only sixth-year data, logistic regression analysis (backward LR) was conducted to identify independent factors associated with psychological distress, depression, and anxiety, and three separate models were created. Independent variables that were statistically significant in univariate analyses were included in the models, taking into account potential correlations between them. Age, family type, income level, smoking and alcohol use, presence of psychiatric illness in the family, daily sleep duration, voluntary choice of medical school, presence of a deceased relative or friend during the pandemic, and presence of a relative or friend affected by the earthquake were found to be statistically insignificant in univariate analyses and therefore were not included in the logistic regression model. The independent variables included are described in detail in the relevant part of the results section. In statistical analyses, a *p*-value of <0.05 was considered significant.

## 3. Results

At the beginning of the study, the participants had a mean age of 18.3 ± 0.7 years, and 50.4% were female; changes over time are presented in Table 1. Among the students who participated in the study, 85.7% came from nuclear families, and 73.0% had families living in a different city. For 31.8% of the students, family income exceeded expenses, while for 59.1%, income and expenses were equal. The frequency of mothers who were college or university graduates was 36.5%, and for fathers, this rate was 52.8%. A total of 90.9% of the students had chosen to study medicine willingly, while the rest had done so due to external guidance.

When the baseline mental health scores (GHQ, BDI, STAI, IAT) of students who completed all three time points (*n* = 230) were compared with those of students who dropped out (*n* = 52), no statistically significant differences were found between the two groups (*p* < 0.05).

The mean, standard deviation, and median scores obtained from the scales used in the study, as well as their changes over time, are presented in Table 2.

The median GHQ score was found to be increased significantly in the third and sixth years compared to baseline (*p* < 0.001). While no significant difference was observed in the median BDI and S-Anxiety scores between baseline and the third year, an increase was found between the third and sixth years as well as between baseline and the sixth year. Although the median T-Anxiety score increased from baseline to the sixth year, the difference was not statistically significant (*p* = 0.153). A significant increase over the years was observed in the median IAT score (*p* < 0.001) (Table 2).

Based on the cut-off points defined in the Turkish validity and reliability studies, scores from the scales were converted into categorical variables to determine the prevalence of psychological distress, depressive symptoms, and S-Anxiety, and the changes over time were compared (Table 3).

Although the frequency of students who scored 2 or above on the GHQ scale increased over the years, the increase was not statistically significant (*p* = 0.210). According to the BDI scale, no difference was found in the frequency of students showing depressive symptoms between baseline and the third year, while an increase was observed between the third and sixth years (*p* = 0.010). The prevalence of S-Anxiety also increased significantly from the third to the sixth year (*p* = 0.001). According to the IAT scale, the frequency of risky internet use increased significantly from the third to the sixth year (*p* = 0.010) (Table 3). In all three years, no students scored 80 or above, the threshold for being classified as internet-addicted.

Some life events considered potential sources of stress were assessed, and those that showed significant changes over time are presented in Table 4 and Table 5.

Among the potential sources of stress, personal and societal concerns about the future increased significantly from baseline to the third year and from the third to the sixth year (*p* < 0.01). Financial problems and high parental expectations were also found to increase from the third to the sixth year (*p* < 0.01) (Table 4).

“Dissatisfaction with occupational choice” and “concern about the inadequacy of the education received” showed a significant increase over the years (*p* < 0.001). Factors asked only in the third and sixth years (the process of preparing for the Medical Specialty Examination, mandatory service requirement after graduation, and negative attitudes from faculty members) emerged as increasingly significant issues for students during the clinical years (*p* < 0.001) (Table 5). For other potentially stress-inducing events such as dissatisfaction with social life, communication problems with the opposite sex, and perceived academic success concerns, no statistically significant difference was found over the years (*p* > 0.05).

To determine the factors affecting students’ psychological distress, logistic regression analysis was performed using sixth-year data, including variables that were found to be statistically significant in univariate analyses. These variables were gender, doing regular exercise, education level of the father, being diagnosed with COVID-19, experiencing economic problems, high parental expectations, societal concerns about the future, communication problems with the opposite sex, negative attitudes from faculty members, preparation for the Medical Specialty Examination, mandatory service, dissatisfaction with social life, dissatisfaction with occupational choice, perceived academic success concerns, and concern about the adequacy of the education received. As a result of the analysis, the variables found to be risk factors for psychological distress among medical students were having a father who is a university graduate, having been diagnosed with COVID-19 during the pandemic, having societal concerns about the future, being obligated to undertake mandatory service, dissatisfaction with social life, and dissatisfaction with career choice (GHQ-12 Nagelkerke R^2^ = 0.445) (Table 6).

To determine the factors affecting the presence of depressive symptoms, the following variables were included in the logistic regression analysis: education level of the father, having been diagnosed with COVID-19, high parental expectations, concerns about societal future, mandatory service, dissatisfaction with social life, dissatisfaction with occupational choice, perceived academic success concerns, concern about the adequacy of the education received, and risky internet use. Although gender was not found to be statistically significant in univariate analyses, it was included in the model based on its relevance in the literature. It was determined that students whose fathers were university graduates, who had been diagnosed with COVID-19, who used the internet at a risky level, who had high parental expectations, who were dissatisfied with their social life, and who had greater academic performance anxiety were at increased risk for depressive symptoms (BDI Nagelkerke R^2^ = 0.308) (Table 6).

To identify the factors affecting students’ S-Anxiety, the following variables were included in the model: gender, having been diagnosed with COVID-19, high parental expectations, communication problems with the opposite sex, negative attitudes from faculty members, preparation for the Medical Specialty Examination, mandatory service, dissatisfaction with social life, dissatisfaction with occupational choice, perceived academic success concerns, concern about the adequacy of the education received, and risky internet use. It was found that students with high parental expectations, those dissatisfied with their occupational choice, those concerned about the adequacy of their education, and those experiencing communication problems with the opposite sex were at risk for S-Anxiety (S-Anxiety Nagelkerke R^2^ = 0.302) (Table 6).

## 4. Discussion

In this study, students who started their education at a medical faculty were monitored throughout their education in terms of mental health. Data collected at the start of medical school were recollected at the end of the third and sixth years, and statistical analyses were conducted. A review of the literature revealed that there are few longitudinal studies on mental health problems among medical students globally, and most have had relatively short durations [7,14,28,29,30,31,32]. Additionally, differences in the psychometric tools used and the cut-off points applied across countries make the comparison of findings challenging for discussion. To the best of our knowledge, this is the first six-year longitudinal study conducted in Türkiye on this subject among medical students.

Although the mean scores on the GHQ-12, which indicates the presence of psychological distress, showed a statistically significant increase over time, the prevalence based on the cut-off point did not increase significantly. According to our findings, more than half of the students experienced psychological distress when starting medical school. Some studies suggest that mental disorders begin during adolescence. A systematic review found that the prevalence of psychological distress among adolescents globally was 31.0% and 25.0% when GHQ-12 cut-off points of 3 and 4 were used, respectively [33]. In our study, we used the cut-off point of 2, as specified in the Turkish validation study, and the prevalence of psychological distress was found to be 53.9%. This suggests that psychological distress may begin during adolescence, even before students experience the challenges of medical education. In Türkiye, gaining admission to medical school requires intensive study and a high score on a competitive entrance exam, which may create significant psychological pressure. No six-year longitudinal study on medical students using the GHQ-12 was found in the literature. In a five-year follow-up study at another medical school, a decline in the prevalence of psychological distress was observed in the fourth and fifth years, unlike our findings, though this decrease was not statistically significant [29]. In a study conducted at the nursing faculty of the same university in the present study, the prevalence of psychological distress increased significantly from 42.2% in the first year to 56.5% in the final year [34]. Similarly, a longitudinal study conducted among dental students in Jordan reported a statistically significant increase in psychological distress from an initial rate of 58% [35]. These findings suggest that not only medical students but also students in other health-related fields may be psychologically affected during their training.

When analyzing median BDI scores and the prevalence of depressive symptoms based on the cut-off point, a statistically significant increase from the first to the sixth year was observed. Although different studies have used various scales, several have also reported increases in depression rates among medical students from the first to the second or third year [7,36,37]. A study at the same university’s nursing faculty showed an increase in depressive symptoms from the first to the fourth year, although this increase was not statistically significant [34]. In contrast to our findings, a longitudinal study by Silva et al. found that both BDI scores and depression prevalence decreased during medical education [31]. A meta-analysis of cross-sectional studies reported that depression was most common among first-year medical students and tended to decline over time, with approximately one-third of medical students affected globally [11]. A systematic review found that the global average prevalence of depression among medical students was 27.0% (95% CI: 24.7–29.5), with a rate of 20.1% in the European region [38]. In our study, the prevalence of depression more than doubled from baseline to the sixth year, reaching 21.3%. While no significant difference was observed between baseline and the third year, a significant increase was found between the third and sixth years. This indicates that the mental health of our students was adversely affected during the clinical training period. The COVID-19 pandemic, which began during the fourth year of their education, may have contributed to this increase. A systematic review and meta-analysis emphasized that a significant proportion of medical students were psychologically affected by the pandemic, with elevated risks of anxiety and depression development compared to pre-pandemic levels [39].

Median S-Anxiety scores and the prevalence of S-anxiety based on cut-off levels increased significantly from the third to the sixth year, as well as from the first to the final year. A similar trend was observed in a study of nursing students at the same university, where both the scores and prevalence of state anxiety increased significantly from the first to the fourth year [34]. In a study conducted 20 years ago at the same medical school, Aktekin et al. found a statistically significant increase in S-Anxiety scores from the first to the second year [7]. A six-year longitudinal study in Germany reported significant increases in stress, anxiety, and depression from the first to the second year, followed by decreases in subsequent years [14]. In Brazil, a three-year follow-up study found increases in depression, anxiety, and stress scores from the first to the third year [40]. However, a meta-analysis of cross-sectional studies showed that while anxiety prevalence was slightly higher among clinical students than in preclinical students, the difference was not statistically significant [12].

Although the median T-Anxiety score increased from baseline to the sixth year in our study, the difference was not statistically significant. A study conducted 20 years ago at the same faculty found a significant increase in T-Anxiety scores from the first to the second year [7]. A study with nursing students found a significant increase in T-Anxiety scores from the first to the final year [34].

A significant increase over the years was found in the median IAT score, used to assess internet addiction. Based on the cut-off points, the frequency of risky internet use increased significantly from the first to the sixth year. Approximately 15% of final-year students were classified as risky internet users. A study on nursing students also found a significant increase in risky internet use from the first to the final year, with a frequency similar to that in our study [34]. In another three-year follow-up study initiated in the same year as ours, the prevalence of internet addiction among medical students increased from the first to the third year, with nearly half of third-year students classified as addicted [41]. According to a meta-analysis, the prevalence of internet addiction among medical students is approximately 26.0% [6]. Internet addiction has been shown to be associated with psychological, physical, and social problems, including poor academic performance, and to have negative mental health outcomes in medical students [42]. These findings highlight the growing problem of internet addiction among students and the need to address it.

In our study, it was observed that 90.9% of the students chose medical school willingly in the university entrance exam, but their dissatisfaction with occupational choices increased over the years. Medical education is long, demanding, and stressful, both physically and mentally. Encountering patients and their relatives during clinical rotations, especially during final-year night shifts, taking on caregiving responsibilities, and facing patient deaths may contribute to the psychological distress of students. However, these challenges may not be solely attributed to the difficulty of medical education. The healthcare system structure in Türkiye also poses challenges. Every medical graduate is required to undertake mandatory service or pass the national Medical Specialty Examination to become a specialist, after which they are again obligated to complete a period of mandatory service. Therefore, in the final years of education, concerns about the future are added to academic burden and patient care responsibilities. This may explain the significant increase in both personal and societal future concerns among our students. A qualitative study in Türkiye found that final-year medical students had serious concerns about their professional training and post-graduation careers [43]. A cross-sectional study involving final-year students from 39 different medical faculties in Türkiye reported that 70.7% intended to relocate abroad after graduation, citing poor working conditions and doubts about the quality of education as key reasons [44]. In our study, concern about inadequate education significantly increased over the years and was also found to be a risk factor for state anxiety.

According to logistic regression analysis based on sixth-year data, students whose fathers were university graduates, who had been diagnosed with COVID-19, and who were dissatisfied with their social lives were found to be at increased risk for psychological distress and depression. Students with high parental expectations were found to be at risk of depression and S-anxiety. Those dissatisfied with their occupational choice were at risk for both psychological distress and S-anxiety. These findings are consistent with previous studies [10,45]. Considering that dissatisfaction with social life is a risk factor for both psychological distress and depression, the academic curriculum should be reviewed to reduce its intensity and allow students more free time. Our faculty is located on one of the largest university campuses in Türkiye, which offers numerous activity areas. Creating time for students to take advantage of these facilities could be a significant step toward protecting their mental health.

The findings of the study contribute to the Sustainable Development Goals, which aim to “ensure healthy lives and promote well-being for all at all ages” [46]. The research results also raise awareness of the development/support of the health workforce.

A major strength of our study is its six-year longitudinal design. As for limitations, the study was only conducted in a single medical school, and the conclusions may not be generalizable to medical students in other regions or with different cultural backgrounds. External events such as the COVID-19 pandemic and earthquakes occurred during the study period, which may have influenced the results. The loss rate at the sixth-year follow-up was 18.4%. This may have caused the results/psychological changes we found to appear less significant than they actually were.

## 5. Conclusions

In conclusion, it was found that the mental health of medical students deteriorated over the course of their education, especially during the clinical years. As these future physicians will be responsible for protecting and improving public health, it is essential to prioritize the mental well-being of medical students. It is important to develop intervention programs to protect the psychological health of students, especially during the clinical years of education. During this period, the frequency of consultant/mentor follow-ups can be increased through structured forms. Students identified as needing assistance can be quickly reported to the term coordinators. Regular meetings can be held where term or internship student representatives, term coordinators, and faculty administrators come together. To protect the psychological health of students, the curriculum should be adjusted to allow for more free time, and participation in social, cultural, and sports activities should be encouraged so students can be with their peers. Protecting the mental health of medical students will be beneficial not only for the students themselves but also for the patients and communities they will serve in the future.

## Figures and Tables

**Table 1:** Descriptive characteristics of medical students by year.

Demographic Characteristics	Baseline	3rd Year	6th Year
Participants (*n*)	282	259	230
Response/follow-up rate (%)	100.0	91.8	81.6
Age (Mean ± SD) (minimum–maximum)	18.3 ± 0.7 (17–22)	20.8 ± 0.9 (19–25)	24.1 ± 0.9 (23–27)
Sex			
Female	142 (50.4)	136 (52.5)	122 (53.0)
Male	140 (49.6)	123 (47.5)	108 (47.0)

SD, standard deviation.

**Table 2:** The scores of scales for medical students and their changes over time.

Scales (min–max)	Periods	Values		χ^2^/Z	*p*
		Mean ± SD	Median (min–max)		
GHQ-12 (0–36)	Baseline	11.17 ± 5.19	10.50 (0–27)	42.055 *	<0.001
	3rd year	12.47 ± 5.76	12.00 (0–36)		
	6th year	14.09 ± 6.35	13.00 (0–36)		
		Baseline and 3rd year		3.631 **	<0.001
		3rd year and 6th year		3.644 **	<0.001
		Baseline and 6th year		5.781 **	<0.001
BDI (0–63)	Baseline	7.83 ± 6.46	6.00 (0–36)	12.888 *	0.002
	3rd year	8.73 ± 7.21	7.00 (0–41)		
	6th year	10.54 ± 9.27	9.00 (0–62)		
		Baseline and 3rd year		1.879 **	0.060
		3rd year and 6th year		2.874 **	0.004
		Baseline and 6th year		3.824 **	<0.001
S-Anxiety (20–80)	Baseline	38.33 ± 9.86	37.50 (20–75)	17.464 *	<0.001
	3rd year	38.87 ± 9.67	38.00 (20–74)		
	6th year	42.27 ± 10.97	42.00 (20–76)		
		Baseline and 3rd year		1.029 **	0.304
		3rd year and 6th year		4.186 **	<0.001
		Baseline and 6th year		4.334 **	<0.001
T-Anxiety (20–80)	Baseline	43.09 ± 8.77	43.00 (22–70)	3.758 *	0.153
	3rd year	43.25 ± 9.53	42.00 (24–73)		
	6th year	44.50 ± 10.33	45.00 (20–74)		
IAT (0–100)	Baseline	26.70 ± 14.30	26.00 (0–90)	16.629 *	<0.001
	3rd year	29.13 ± 14.49	27.00 (3–78)		
	6th year	31.78 ± 16.36	30.50 (0–73)		
		Baseline and 3rd year		2.513 **	0.012
		3rd year and 6th year		2.391 **	0.017
		Baseline and 6th year		4.506 **	<0.001

Abbreviations: GHQ-12, General Health Questionnaire; BDI, Beck Depression Inventory; S-Anxiety, The State Anxiety Scale; T-Anxiety, The Trait Anxiety Scale; IAT, Internet Addiction Test; SD, standard deviation. * Friedman Test, ** Wilcoxon Signed Ranks Test.

**Table 3:** Prevalences of scores on scales among medical students and their changes over time.

Scales	Periods	Values		Cochran’s Q	*p*
GHQ-12		0–1 point *n* (%)	≥2 points *n* (%)		
	Baseline	106 (46.1)	124 (53.9)	3.122	0.210
	3rd year	98 (42.6)	132 (57.4)		
	6th year	90 (39.1)	140 (60.9)		
BDI		0–16 points *n* (%)	≥17 points *n* (%)		
	Baseline	209 (90.9)	21 (9.1)	17.268	<0.001
	3rd year	200 (87.0)	30 (13.0)		
	6th year	181 (78.7)	49 (21.3)		
		Baseline and 3rd year		1.561 *	0.212
		3rd year and 6th year		6.612 *	0.010
		Baseline and 6th year		14.019 *	<0.001
S-Anxiety		0–39 points *n* (%)	≥40 points *n* (%)		
	Baseline	130 (56.5)	100 (43.5)	17.339	<0.001
	3rd year	126 (54.8)	104 (45.2)		
	6th year	95 (41.3)	135 (58.7)		
		Baseline and 3rd year		0.122 *	0.727
		3rd year and 6th year		10.345 *	0.001
		Baseline and 6th year		12.430 *	<0.001
IAT		0–49 points *n* (%)	≥50 points *n* (%)		
	Baseline	216 (93.9)	14 (6.1)	14.735	0.001
	3rd year	211 (91.7)	19 (8.3)		
	6th year	195 (84.8)	35 (15.2)		
		Baseline and 3rd year		0.696 *	0.405
		3rd year and 6th year		6.618 *	0.010
		Baseline and 6th year		9.756 *	0.002

Abbreviations: GHQ-12, General Health Questionnaire; BDI, Beck Depression Inventory; S-Anxiety, The State Anxiety Scale; IAT, Internet Addiction Test. * McNemar’s test.

**Table 4:** Changes in potential stress-inducing factors over the years.

Causes of Stress	Periods	Values (Minimum = 0–Maximum = 10)		χ^2^/Z	*p*
		Mean ± SD	Median (min–max)		
Financial problems	Baseline	3.17 ± 2.35	3.00 (0–10)	132.326 *	<0.001
	3rd year	3.55 ± 2.67	3.00 (0–10)		
	6th year	5.64 ± 2.71	6.00 (0–10)		
		Baseline and 3rd year		2.016 **	0.044
		3rd year and 6th year		8.911 **	<0.001
		Baseline and 6th year		10.071 **	<0.001
Concerns about the future—individual	Baseline	4.05 ± 2.71	4.00 (0–10)	107.727 *	<0.001
	3rd year	4.73 ± 2.84	5.00 (0–10)		
	6th year	6.57 ± 2.78	7.00 (0–10)		
		Baseline and 3rd year		3.171 **	0.002
		3rd year and 6th year		7.989 **	<0.001
		Baseline and 6th year		9.559 **	<0.001
Concerns about the future—societal	Baseline	5.17 ± 3.09	5.00 (0–10)	99.726 *	<0.001
	3rd year	6.27 ± 3.05	6.50 (0–10)		
	6th year	7.55 ± 2.58	8.00 (0–10)		
		Baseline and 3rd year		4.906 **	<0.001
		3rd year and 6th year		5.907 **	<0.001
		Baseline and 6th year		8.999 **	<0.001
High parental expectations	Baseline	2.94 ± 2.79	2.00 (0–10)	13.727 *	0.001
	3rd year	2.83 ± 2.45	2.00 (0–10)		
	6th year	3.43 ± 2.72	3.00 (0–10)		
		Baseline and 3rd year		0.580 **	0.562
		3rd year and 6th year		3.512 **	<0.001
		Baseline and 6th year		2.371 **	0.018

Abbreviations: SD, standard deviation. * Friedman test, ** Wilcoxon signed ranks test.

**Table 5:** Changes over the years in medical-education-related potential factors that may cause stress.

Causes of Stress	Periods	Values (Minimum = 0–Maximum = 10)		χ^2^/Z	*p*
		Mean ± SD	Median (min–max)		
Dissatisfaction with occupational choice	Baseline	1.36 ± 1.94	1.00 (0–10)	168.081 *	<0.001
	3rd year	2.74 ± 2.48	2.00 (0–10)		
	6th year	4.36 ± 2.84	4.00 (0–10)		
		Baseline and 3rd year		7.486 **	<0.001
		3rd year and 6th year		7.553 **	<0.001
		Baseline and 6th year		10.472 **	<0.001
Concern about the inadequacy of the education received	Baseline	1.95 ± 2.14	1.00 (0–9)	130.863 *	<0.001
	3rd year	3.42 ± 2.63	3.00 (0–10)		
	6th year	4.78 ± 2.83	5.00 (0–10)		
		Baseline and 3rd year		7.370 **	<0.001
		3rd year and 6th year		6.156 **	<0.001
		Baseline and 6th year		10.182 **	<0.001
Process of preparing for the Medical Specialty Examination	Baseline	Not collected		9.435 **	<0.001
	3rd year	3.52 ± 3.30	3.00 (0–10)		
	6th year	6.67 ± 2.97	7.00 (0–10)		
Mandatory service requirement after graduation	Baseline	Not collected		9.744 **	<0.001
	3rd year	4.16 ± 3.23	4.00 (0–10)		
	6th year	7.00 ± 3.02	8.00 (0–10)		
Negative attitudes from faculty members	Baseline	Not collected		8.121 **	<0.001
	**3rd year**	1.60 ± 2.09	1.00 (0–10)		
	**6th year**	3.40 ± 2.85	3.00 (0–10)		

Abbreviations: SD, standard deviation. * Friedman test. ** Wilcoxon signed ranks test.

**Table 6:** Results of logistic regression analyses for medical students.

Scale	Variables	B	SE	OR	95% CI	*p*
GHQ-12 ≥2 points	Father being a university graduate	1.150	0.354	3.157	1.578–6.314	0.001
	Being diagnosed with COVID-19	1.042	0.427	2.835	1.226–6.552	0.015
	Societal concerns about the future	0.179	0.073	1.196	1.036–1.381	0.014
	Being obligated to undertake mandatory service	0.228	0.065	1.256	1.106–1.426	<0.001
	Dissatisfaction with social life	0.278	0.073	1.320	1.144–1.523	<0.001
	Dissatisfaction with occupational choice	0.219	0.069	1.245	1.088–1.424	0.001
	Nagelkerke R^2^ = 0.445					
BDI ≥17 points	Father being a university graduate	1.271	0.405	3.565	1.612–7.888	0.002
	Being diagnosed with COVID-19	0.796	0.401	2.217	1.011–4.865	0.047
	High parental expectations	0.135	0.066	1.145	1.005–1.303	0.042
	Dissatisfaction with social life	0.155	0.072	1.168	1.014–1.345	0.031
	Perceived academic success concerns	0.172	0.068	1.188	1.040–1.357	0.011
	Risky internet use	0.973	0.438	2.645	1.121–6.237	0.026
	Nagelkerke R^2^ = 0.308					
S-Anxiety ≥40 points	High parental expectations	0.177	0.061	1.193	1.059–1.344	0.004
	Dissatisfaction with occupational choice	0.174	0.059	1.191	1.060–1.338	0.003
	Worry about the adequacy of education	0.138	0.062	1.148	1.017–1.297	0.025
	Communication problems with the opposite sex	0.152	0.071	1.165	1.013–1.340	0.033
	Nagelkerke R^2^ = 0.302					

Abbreviations: GHQ-12, General Health Questionnaire; BDI, Beck Depression Inventory; S-Anxiety, The State Anxiety Scale; R^2^, R squared; SE, standard error; OR, odds ratio; CI, confidence interval.

## Data Availability

Data are available upon request from the corresponding author.
